# Effect of Octreotide on Enteric Motor Neurons in Experimental Acute Necrotizing Pancreatitis

**DOI:** 10.1371/journal.pone.0052163

**Published:** 2012-12-26

**Authors:** Hui Zhou, Jun Gao, Duowu Zou, Wenbin Wu, Zhaoshen Li

**Affiliations:** 1 Department of Gastroenterology, Shanghai First People's Hospital, School of Medicine, Shanghai Jiaotong University, Shanghai, China; 2 Division of Gastroenterology, Department of Internal Medicine, Changhai Hospital, Second Military Medical University, Shanghai, China; 3 Laboratory of Stress Research, Department of Internal Medicine, Changhai Hospital, Second Military Medical University, Shanghai, China; University of Medicine & Dentistry of NJ - New Jersey Medical School, United States of America

## Abstract

**Background/Aims:**

Amelioration of intestinal dysmotility and stasis during the early period of acute necrotizing pancreatitis (ANP) appears to be important to reduce the risks of secondary pancreatic infection. We aimed to characterize the association between the neuropathy of the enteric nervous system and gut dysfunction and to examine the effect of octreotide on motor innervation in the early stage of ANP.

**Methodology/Principal Findings:**

The rats were randomly divided into eight groups: control+saline; control+octreotide; ANP+saline and ANP+octreotide (24 h, 48 h, 72 h). The spontaneous activity of ileal segments and the response to ACh, l-NNA were recorded. The alterations of myenteric neuronal nitric oxide synthase (nNOS), choline acetyltransferase (CHAT), PGP9.5 and somatostatin receptor 2 (SSTR_2_) immunoreactive cells were evaluated by immunofluorescence and the protein expression of nNOS and CHAT were evaluated by western blot. We found the amplitude of spontaneous contractions at 48 h and the response to ACh at 24 h declined in the ANP+saline rats. A higher contractile response to both ACh and to l-NNA was observed in the ANP+octreotide group, compared with the ANP+saline rats at 24 h. A significant reduction in the nNOS and cholinergic neurons was observed in ANP+saline rats at the three time points. However, this reduction was greatly ameliorated in the presence of octreotide at 24 h and 48 h. The protein expression of CHAT neurons at 24 h and the nNOS neurons at 48 h in the ANP+octreotide rats was much higher than the ANP+saline rats.

**Conclusion:**

The pathogenesis of ileus in the early stage of ANP may be related to the neuropathy of the enteric nervous system. Octreotide may reduce the severity of ileus by lessening the damage to enteric motor innervation.

## Introduction

Pancreatic infection associated with acute necrotizing pancreatitis (ANP) has emerged as the most important determinant for late morbidity and mortality from this serious disease [1], [2]. The current hypothesis is that this infection originates from the gut. In healthy subjects, reciprocal regulatory influences exist between the intestinal microflora and small bowel motility [3]. In patients with severe acute pancreatitis (SAP), the symptoms of flatulence and abdominal distention, nausea, and vomiting related to the disturbed gastrointestinal motility are usually observed. It is postulated that ileus with bacterial overgrowth is a major player in the pathogenesis of pancreatic infection [4], [5], [6], [7]. Therefore, amelioration of intestinal dysmotility and stasis during the early period of ANP appears to be important to reduce the risks associated with these serious complications.

It is well known that gastrointestinal motor events are modulated by highly complex and integrated systems including neural, myogenic, and local hormonal regulation. Dysmotility in a certain of pathological state is probably associated with muscular and neural damage. The pathogenic mechanisms of ileus in SAP are largely unknown. Our previous research has shown that the pathogenesis of small intestinal paralysis in rats with ANP may be related to deficiencies in neuromuscular function [8], [9]. Examination of neural–immune interactions suggests that inflammation-associated damage of the enteric nervous system (ENS) may cause gastrointestinal dysmotility [10], [11], [12], [13], [14]. The observed changes in both inhibitory and excitatory enteric motor neurons have shed light on the mechanisms mediating disturbances of motility [10], [11], [12]. Neuroplastic change initiated by an inflammatory insult may be the major precipitating factor leading to structural damage of the ENS as well as up- or down-regulation of receptor systems.

Somatostatin (SOM) inhibits the release of growth hormone, blocks the exocrine function of the stomach and the pancreas, regulates peristalsis, and modulates enteric neurotransmission [15]. Octreotide, the long-acting SOM analogue with selectivity for SOM receptor 2 (SSTR_2_) [16], has been used in many tertiary centers to treat SAP because of its inhibitory effect on pancreatobiliary secretion. Octreotide has also been reported to have the ability of direct anti-inflammation and regulating the gastrointestinal motility in both animals and humans [15], [16], [17]. A variety of cytokines are involved in the pathophysiological process of SAP [18], [19]. Octreotide has obvious protective effects on the multiple organ injury via a mechanism that is associated with the inhibition of inflammatory mediators [20], [21], [22], [23], [24]. From a physiological perspective, the peripheral inhibitory effect of octreotide or SOM on intestinal peristalsis is well documented [25], [26], [27]. Experiments in a variety of laboratory animals suggest that SOM excites enteric neurons mediating relaxation and inhibits neurons mediating contraction of the external muscle within enteric circuits that organize the timing of motor activity [28], [29], [30], [31]. The interplay of SOM and its responsive nitrergic and cholinergic innervation has been implicated in the pathway of enteric regulation [29]. However, how a substance works in physiological states does not necessarily predict its effect in pathological conditions, such as inflammation.

In the current study, we aimed to characterize the neuropathy of the ENS related to gut dysfunction after the onset of ANP and to examine the effect of octreotide on the alterations of the neuroregulatory circuit in the ENS under the conditions imposed by ANP.

## Materials and Methods

### Materials and chemicals

Acetylcholine chloride (ACh) was obtained from Shanghai URChem (Shanghai, China) and N^G^-nitro-l-arginine (l-NNA) was obtained from Sigma-Aldrich (St. Louis, MO). All other materials and chemicals were obtained from Shanghai Yuanyuan Chemical Reagent (Shanghai, China).

### Animals

Male Sprague–Dawley rats weighing about 200–300 g were used. The animals were housed in individual cages with free access to water and food. Housing conditions were kept constant: temperature 22°C, relative humidity 40%, and a 12-h light/dark cycle. The animals were allowed to adjust to these conditions for 1 week before surgery. All procedures received approval from the Administrative Committee of Experimental Animal Care and Use of the Second Military Medical University, Shanghai, China.

### Model for acute necrotizing pancreatitis

The rats in pancreatitis group were injected with l-ornithine, in accordance with the procedure of Rakonczay et al [32]. In brief, ANP was induced in these rats by intraperitoneal injections of 30% l-ornithine at a dose of 3 g/kg at hourly intervals. The control rats received injections of normal saline.

### Immunohistochemistry

For immunohistochemistry, whole-mount preparations were used as described previously [33]. Immunohistochemical staining was performed against PGP9.5, choline acetyltransferase (CHAT), neuronal nitric oxide synthase (nNOS), and somatostatin receptor 2 (SSTR_2_), as the markers of enteric nerves, CHAT, nNOS, and SSTR_2_ immunoreactive myenteric nerves, respectively. The ileum was inflated by injecting paraformaldehyde into the lumen, and both ends were ligated with cotton threads. These segments were dissected, removed, and then immersed in paraformaldehyde for 4–6 h at 4°C. The lumen was opened and the mucosa and submucosal layers were peeled off with forceps under a dissection microscope. The circular muscle layer and the myenteric region were separated by sharp dissection with forceps under a dissection microscope. The myenteric region specimens were subsequently immersed in 0.3% Triton X-100 in phosphate-buffered saline (PBS) for 2 h and then blocked with 3% BSA–PBS for 1 h.

For the PGP9.5, nNOS, and SSTR_2_ staining, the myenteric region specimens were incubated with 0.3% Triton X-100 in 10% normal goat serum for 60 min and incubated in rabbit anti-PGP9.5 polyclonal antibody (sc-25800; 1∶200 in 0.05 M PBS; Santa Cruz Biotechnology, Santa Cruz, CA) or rabbit anti-nNOS antibody (Catalog #07-571; 1∶400 in 0.05 M Tris buffer; EMD Millipore, Billerica, MA) or rabbit anti-SSTR_2_ antibody (sc-25676; 1∶50 in 0.05 M PBS; Santa Cruz Biotechnology) at 4°C overnight. After a thorough wash with PBS, the specimens were labeled with FITC-conjugated secondary antibody (goat anti-rabbit IgG, 1∶200 in 0.05 M Tris buffer; Molecular Probes, Invitrogen, Life Technologies, Grand Island, NY) at room temperature for 1 h and then rinsed in PBS three times for a total 30 min.

For the CHAT staining, the myenteric region specimens were incubated with 0.3% Triton X-100 in 10% normal rabbit serum for 60 min and incubated in goat anti- CHAT polyclonal antibody (Catalog #AB144P; 1∶100 in 0.05 M PBS; EMD Millipore) at 4°C overnight. After a thorough wash with PBS, they were labeled with Cy3-conjugated secondary antibody (rabbit anti- goat IgG, 1∶200 in 0.05 M Tris buffer; Molecular Probes) at room temperature for 1 h and then rinsed three times in PBS for a total 30 min.

All secondary antibodies were purchased from Jackson Immunoresearch Laboratories (West Grove, PA). Finally, the specimens were mounted on glass slides and coverslipped. For control experiments, either the primary or the secondary antibodies were omitted. All the immunolabeled specimens were observed with a IX71 fluorescence microscope (Olympus, Japan). For the observations of single-immunolabeled specimens by either Cy3 or FITC, the excitation wavelengths of laser light were adjusted to 552 nm for Cy3 and to 488 nm for FITC, respectively.

### In vitro study of small intestine motility

Control rats and ANP rats (eight groups) were used for in vitro experiments. The motility of isolated small intestine segments was measured as reported previously [34]. The rats were anesthetized with 3% soluble pentobarbitone (30 mg/kg) and exsanguinated. The small intestines were removed quickly, gently flushed, and placed into a cold aerated HEPES-buffered physiological solution (composition in mM: NaCl 126, KCl 6, MgCl_2_ 1.2, CaCl_2_ 2, EDTA 0.01, HEPES 10.5, and glucose 14, pH 7.4).

The organ bath technique was performed using full-thickness intestinal segments. 1-cm segments of small intestine were placed vertically in a 10-mL bath filled with HEPES-buffered physiological solution. The solution was maintained at 37°C and aerated with a mixture of 95% O_2_ and 5% CO_2_. Each intestinal segment was positioned between the two iron rings mounted on an iron rod. The lower end of the intestinal segment was fixed to the rod and the upper end was connected to a strain gauge transducer for continuous recording of isometric tension.

After an initial equilibration period of 20 min, the intestinal segment was adjusted to maintain 1 g of stable tension at the beginning of experiment. The intestinal segment was then allowed to equilibrate for 60 min before experimentation. During the second equilibration period, the intestinal segment was washed every 15 min with fresh HEPES-buffered physiological solution. The amplitude and frequency, as well as area under the curve of ileum contraction were recorded for later analysis.

After the experiments, each intestinal segment was measured (length), blotted dry, and weighed to normalize recording for tissue wet weight. Tension was determined using the method of Rickenbacher [35] and contractions between the different intestinal segments were normalized by converting grams of contraction to grams per square millimeter per section of tissue. The conversion was derived by determining the cross-sectional area using the following equation: (muscle density assumed to be 1.05 mg/mm^3^): mm^2^ = [wet muscle weight (mg)/muscle length (mm)×muscle density (mg/mm^3^)].

### Pharmacological studies

All experiments were performed at the optimal length of the intestinal segment adjusted to maintain 1 g of stable tension. Each intestinal segment was allowed to equilibrate for 10 min. In the first series of experiments, the receptor-mediated contractile activities to ACh (100 µM) was studied. Secondly, the NOS inhibitor l-NNA (100 µM) was used to investigate the effect of NO on intestinal motility. The contractile response of smooth muscle was quantified as the ratio of tone (g×time as total area under the contractile curve) measured for a 3-min period before the administration of them to the tone measured after administration.

### Western blot

nNOS and CHAT expression was measured in ileal segments from ANP rats and age- and sex-matched controls. A segment of ileum was cut along the mesenteric axis and the mucosa was removed in an oxygenated (5% CO_2_/95% O_2_) ice-cold HEPES-buffered physiological solution. The tissue was immediately snap frozen in liquid N_2_ and stored at −80°C. After homogenization in lysis buffer, which contained 150 mM NaCl and 10 mM Tris-HCl (pH 7.5) and a protease inhibitor (Pierce Protein Research Products, Thermo Fisher Scientific, Rockford, IL), the lysate was collected and centrifuged at 4°C for 15 min at 12,000 rpm to remove the insoluble material. The protein concentration of the supernatant was measured by spectrophotometry using the BCA protein assay method (Thermo Fisher Scientific). Equal amounts of protein (10 µg) were run in parallel on 10% (for nNOS detection) SDS-polyacrylamide gels with a biotinylated protein standard. The proteins were subsequently transferred to polyvinylidene difluoride (PVDF) membranes. After blocking with dried milk (5% wt/vol), the blots were incubated overnight at 4°C with the primary antibodies rabbit anti-nNOS (Catalog #07-571; 1/20000 in 0.05 M Tris buffer; Millipore) or goat anti-CHAT polyclonal antibody (Catalog #AB144P; 1∶1000 in 0.05 M PBS; Millipore) and subsequently incubated for 2 h at room temperature with the horseradish peroxidase (HRP)-conjugated secondary antibodies raised against rabbit IgG (1∶1000 dilution) or goat IgG (1∶1000 dilution), respectively. Antibody detection was performed with an enhanced chemiluminescence (ECL) Western blotting detection system, (Millipore). The image acquisition was performed by FluorChem FC2 chemiluminescent, fluorescent, and visible light gel imaging system (Alpha Innotech, R&D Systems, CA). After scanning, the density of the bands corresponded with nNOS (165 kDa) and CHAT (70 kDa) was quantified (in arbitrary units; AU) using ImageJ software (National Institutes of Health, http://rsb.info.nih.gov/ij/download.html). The changes in the expression of nNOS protein, normalized by glyceraldehyde-3-phosphate dehydrogenase (GAPDH), were determined from optical densitometry of immunoblots and shown as relative OD units.

### Experimental procedures

For in vitro study of small intestine motility, the rats were randomly divided into four groups: control+saline; control+octreotide; ANP+saline (24 h, 48 h, 72 h); and ANP+octreotide (24 h, 48 h, 72 h). That was, 8 groups were included in this study (8 rats in each group). The rats of the ANP+saline and ANP+octreotide groups were given ANP induction. The control+saline group and control+octreotide group were induced by IP injections of saline at a dose of 3 g/kg. Six hours after the IP injection of l-ornithine or saline, the rats of control+octreotide group and ANP+octreotide groups received octreotide infusion, while control+saline group and ANP+saline groups received saline. Octreotide (Sandostatin®, Sandoz Pharmaceuticals, NJ) or saline were given by subcutaneous injections (3 µg/kg) and the administration was repeated every 8 h in the following process of observation until 72 h. After the ANP+saline rats and ANP+octreotide rats were sacrificed at 24 h, 48 h, and 72 h, respectively, the control+saline group and control+octreotide group sacrificed at 24 h, segments of terminal ileum were harvested quickly and used for the following experiments. The studies of intestinal motility were performed 24 h after inclusion of the study for control+saline group and control+octreotide group, while 24 h, 48 h, and 72 h after the injection of l-ornithine for ANP+saline groups and ANP+octreotide groups, respectively. For morphological study and Western blot, the same 8 groups were included in this study and performed at the same time points (6 or 5 rats in each group).

### Measurement and statistical analysis

Two intestinal segments from each experimental animal were sampled for immunofluorescent staining. Images of nNOS and CHAT immunoreactive cells were taken in four randomly chosen fields with area 0.2607-mm^2^ (×200 magnification) per whole-mount preparation. The number of nNOS and CHAT immunoreactive cells per myenteric ganglia was calculated directly. All results are shown as mean ± SEM for the number of rats indicated. For statistical analysis, unpaired Student *t* test, paired Student *t* test, and Mann–Whitney *U* test were used to compare the results from control and ANP rats. A *P* value of less than 0.05 (*P*<.05) was considered significant. All data were analyzed with SPSS 16.0 software (SPSS, Chicago, IL).

## Results

### The effect of octreotide on spontaneous contractions after ANP

**Figure 1:**
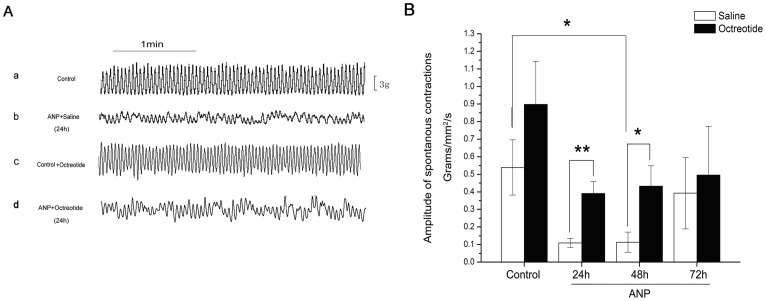
The effect of octreotide on spontaneous contractions of small intestine from ANP rats. The contractile activities were normal in control rats, but were abnormal and variable in ANP rats (*A*). The tendency of decline in amplitude of spontaneous ileal contractions of ANP+saline rats at 48 h was considered to be significant, compared with that in the control+saline rats(*; *P* = .015; n = 8; *B*). Compared with the corresponding ANP+saline group, the amplitude of contractions at 24 h and 48 h were significantly higher in the ANP+octreotide groups (24 h, **, *P* = .001; 48 h, *, *P* = .028; n = 8; *B*). No difference was observed between the 2 groups at the 72 h (*P*>.05, *B*). Data are expressed as means ± SEM. (*, *P*<.05; **, *P*<.01).

In the organ bath experiment, the isometric tension of the isolated segments of distal small intestines from all 8 groups of rats was measured. All segments demonstrated spontaneous contractile activities. The amplitude of the tension was normal in the control rats, but abnormal and variable in the ANP rats (e.g., 24 h after ANP induction, Fig. 1-*A*). The amplitude of spontaneous ileal contractions showed a tendency to decline within 48 h in the ANP+saline group compared with the control+saline group (Fig. 1-*B*). This alteration was considered to be significant at 48 h (control+saline group, 0.5392±0.1572, ANP+saline group, 0.1136±0.0572 g/mm^2^/section; *; *P* = .015; n = 8, Fig. 1-*B*). There was no significant difference in the amplitude of contractions between the ANP+octreotide group and the control+ octreotide group at any of the 3 time points (24 h, 48 h and 72 h, all *P*>.05, n = 8, Fig. 1-*B*). When compared with the ANP+saline group, the amplitude of contractions of the ANP+octreotide group at 24 h and 48 h was much higher (24 h, ANP+saline group, 0.1092±0.0262 g/mm^2^/section; ANP+octreotide group, 0.3910±0.0693 g/mm^2^/section; **; *P* = .001; n = 8; 48 h, ANP+saline group, 0.1136±0.0572 g/mm^2^/section, ANP+octreotide group, 0.4324±0.1164 g/mm^2^/section; *; *P* = .028; n = 8, Fig. 1-*B*). There was no difference between the 2 groups at the 72 h time point (*P*>.05, Fig. 1-*B*).

### The effect of octreotide on pharmacological studies after ANP induction

**Figure 2:**
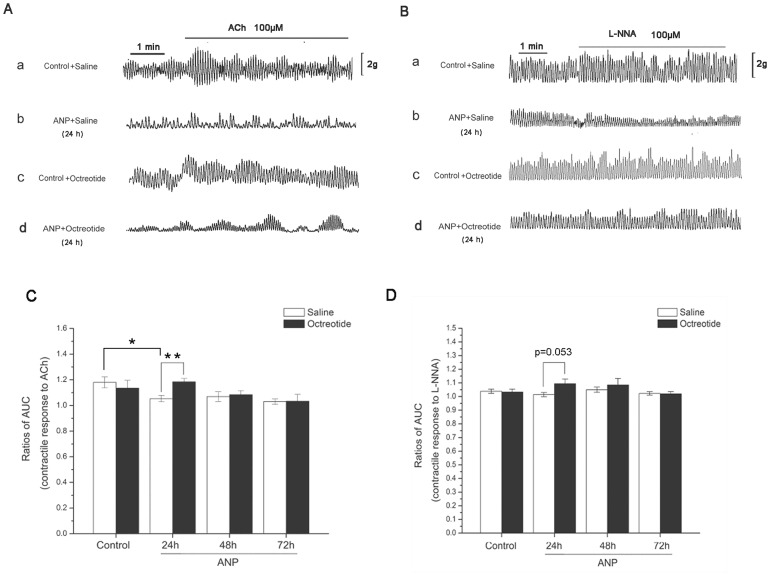
The effect of octreotide on pharmacological studies after ANP. The contractile response to ACh in ileal muscle from the ANP+saline rats were lower than that obtained from the control+saline group at 24 h(*; *P* = 0.026; *A, C*). No significant difference were observed between the ANP+octreotide groups (24 h, 48 h and 72 h) and the control+octreotide group (*C*). The response to ACh in ANP+octreotide group was much higher than the ANP+saline group at 24 h (**; *P* = .002; n = 8; *A, C*). No difference in response to l-NNA was found between any of the ANP+saline groups (24 h, 48 h and 72 h) and the control+saline group or between any of the ANP+octreotide groups (24 h, 48 h and 72 h) and the control+octreotide group (*B, D*). The response to l-NNA in the ANP+octreotide group was higher than the ANP+saline group at 24 h (*P* = .053; n = 8; *B, D*). Data are expressed as means ± SEM. (*, *P*<.05; **, *P*<.01).

In the pharmacological studies, we observed that, compared with the control+saline group, the receptor-mediated contractile response to ACh was significantly decreased in the intestinal muscle from the ANP+saline group at 24 h (control+saline group, 1.1801±0.0422; ANP+saline group, 1.0536±0.0248, *; *P* = 0.026, Fig. 2-*A, C*). The reduction at 48 h and 72 h was not significant. However, there was no significant difference in the contractile responses to ACh between any of the ANP+saline groups (24 h, 48 h and 72 h) and the control+octreotide group. When compared with the corresponding ANP+saline group, the contractile response to ACh in the ANP+octreotide group was much higher at 24 h (ANP+saline group, 1.0536±0.0248; ANP+octreotide group, 1.1849±0.0025; **; *P* = .002; n = 8, Fig. 2-*C*). In the study of the response of contractions to l-NNA, there was no significant difference between any of the ANP+saline groups (24 h, 48 h, 72 h) and the control+saline group (Fig. 2-*B, D*) or between any of the ANP+octreotide groups (24 h, 48 h, 72 h) and the control+octreotide group (Fig. 2-*B, D*). However, between the ANP+octreotide groups and the corresponding ANP+saline groups, the response was similar to that elicited by ACh. The response to l-NNA in the ANP+octreotide group was much higher than in the ANP+saline group at 24 h (1.0951±0.0346 vs 1.0151±0.0154; *P* = .053; n = 8; Fig. 2-*B, D*).

### The effect of octreotide on nNOS, CHAT, PGP9.5, SSTR_2_ immunoreactive myenteric nerve after ANP induction

**Figure 3:**
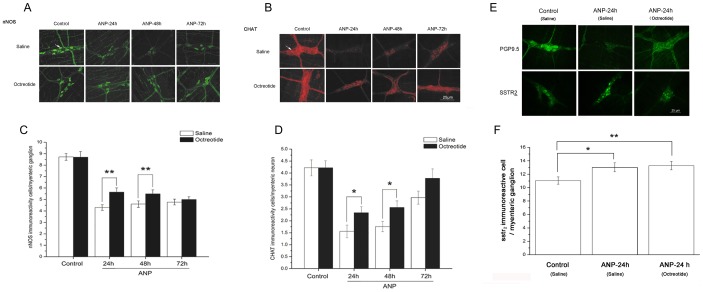
A–D The effect of octreotide on nNOS and CHAT immunoreactive myenteric neurons after ANP. A significant decrease in the number of nNOS immunoreactive cells per myenteric ganglia was evident in all of ANP+saline groups and ANP+octreotide groups, when compared to their controls (for 24 h, 48 h, 72 h, all *P* = .000, not shown; n = 6). The numbers of nNOS immunoreactive cells in the ANP+octreotide groups were much higher than in the corresponding ANP+saline groups at 24 h and 48 h (24 h, **, *P* = .003; 48 h,**, *P* = .0046; n = 6; *A, C*). The CHAT immunoreactive cells per myenteric ganglia significantly decreased in the ANP+saline groups at 3 time points (24 h, *P* = .000; 48 h, *P* = .000; 72 h, *P* = .005; n = 6; *B,D*) and in the ANP+octreotide groups at 24 h and 48 h (24 h, *P* = .000; 48 h, *P* = .000; n = 6; *B,D*) compared with their control. The numbers of CHAT neurons in the ANP+octreotide groups were observed to be higher than in the ANP+saline groups at 24 h and 48 h (24 h, *, *P* = .034; 48 h, *, *P* = .024; n = 6; *B,D*). Data are expressed as means ± SEM. (*, *P*<.05; **, *P*<.01). **E** The effect of octreotide on PGP9.5 and SSTR_2_ immunoreactive myenteric neurons 24 h after ANP. *E* shows the disruption of the myenteric nerve network stained with PGP9.5 in the ANP+saline rats at 24 h. However, the disruption of myenteric ganglia in the ANP+octreotide group appeared to be ameliorated. There was a significant increase in the number of SSTR_2_-positive cells in the ANP+saline rats (*P = .024; n = 6, *F*) and ANP+octreotide at 24 h (**P = .008; n = 6, *F*), compared to the control+saline, respectively. No difference in the numbers of SSTR_2_-positive cells between the ANP+saline and ANP+octreotide groups (*P*>.05, n = 6, *F*). The staining intensity for SSTR_2_ was increased and the diameter of SSTR_2_-positive cells in the ANP+octreotide group was smaller than in the control group. Data are expressed as means ± SEM. (*, *P*<.05; **, *P*<.01).

Fig. 3-*A* shows the distribution of nNOS and CHAT immunoreactivity in all groups. A significant decrease in the number of nNOS immunoreactive cells in the myenteric ganglia was evident in both the ANP+saline and the ANP+octreotide groups, when compared to the control+saline and control+octreotide groups, respectively (for 24 h, 48 h, 72 h; all *P* = .000, n = 6). At 24 h and 48 h, there were many more nNOS immunoreactive cells in the ANP+octreotide groups than in the ANP+saline groups (24 h, ANP+saline, 4.3±0.241; ANP+octreotide, 5.64±0.368, **, *P* = .003; 48 h, ANP+saline, 4.6±0.284, ANP+octreotide, 5.50±0.342, **, *P* = .0046, n = 6; Fig. 3-*A, C*). The morphological observations showed a similar reduction in the number of cholinergic neurons in the ANP+saline groups(24 h, *P* = .000; 48 h, *P* = .000; 72 h, *P* = .005, n = 6) compared with control+saline group, the ANP+octreotide groups at 24 h, 48 h(24 h, *P* = .000; 48 h, *P* = .000, n = 6) but not at 72 h ( *P*>.05, n = 6), compared with their control ( Fig. 3-*B, D*). When compared to each ANP+saline group, CHAT immunoreactive neurons in the ANP+octreotide groups increased at 24 h and 48 h (24 h, ANP+saline, 1.56±0.026, ANP+octreotide, 2.34±0.24, *, *P* = .034; 48 h, ANP+saline, 1.76±0.215; ANP+octreotide, 2.56±0.277, *, P = 0.024; n = 6;Fig. 3-*B, D*) but not at 72 h ( *P*>.05, n = 6) , similar to the results observed with nNOS.

Fig. 3-*E* shows the effect of octreotide on the myenteric nerve network after staining with PGP9.5 24 h after ANP induction. In the control+saline group, a dense network of PGP9.5-positive cells was observed throughout the myenteric plexus. In contrast, the myenteric nerve network of the ANP+saline rats was disrupted and the number of neurons appeared to decrease at 24 h. Compared with the ANP+saline groups, the disruption of the myenteric nerve network and ganglia appeared to be ameliorated in the ANP+octreotide group. There was a significant increase in the number of SSTR_2_-positive cells in the ANP+saline and ANP+octreotide rats at 24 h, compared to the control+saline animals, respectively (ANP+saline, 13.03±0.67, control+saline, 11.05±0.55, *P = .024; ANP+octreotide, 13.28±0.62, control+saline, 11.05±0.55, **P = .008; n = 6, Fig. 3-*F*). There was no difference in the numbers of SSTR_2_-positive cells between the ANP+saline and ANP+octreotide groups (*P*>.05, n = 6, Fig. 3-*F*). In addition, the staining intensity for SSTR_2_ was increased and the diameter of SSTR_2_-positive cells in the ANP+octreotide group was smaller than in the control group.

### Western blot analysis of nNOS

**Figure 4:**
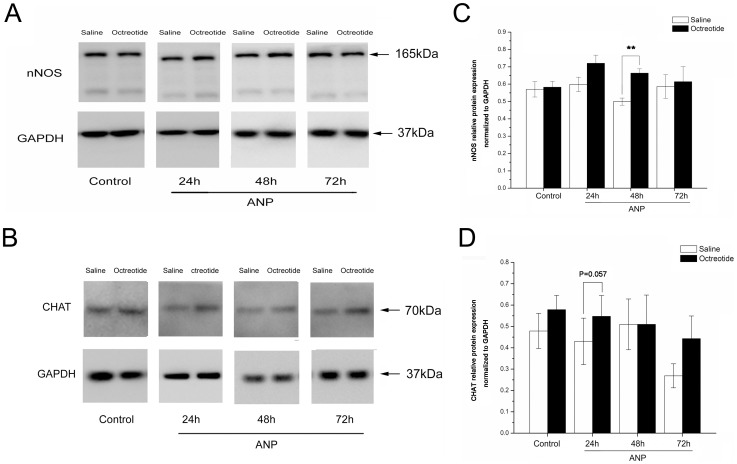
Western blot analysis of nNOS and CHAT. *A* shows relative protein expression of nNOS obtained by densitometric analysis normalized to GAPDH. The predicted molecular weights are indicated by arrows. A drastic difference in protein expression of nNOS between the ANP+saline and ANP+octreotide groups was observed at 48 h (**; *P* = .001; n = 5, *C*). The molecular weight of the lower band corresponds well with the molecular weight of nNOS-ß, a splice variant of nNOS (Ld: protein ladder). *B* shows representative images from CHAT and GAPDH blots. The reduction of the CHAT-IR band density in the ANP+saline rats was more evident than in the ANP+octreotide rats at 24 h, ( *P* = .057; n = 5, *D*). Data are expressed as means ± SEM. (*, *P*<.05; **, *P*<.01).

Western blot analysis using an antibody to nNOS and CHAT on tissue from the small intestine detected protein bands at about 165 kDa and 70 kDa that corresponded to the molecular weights of nNOS protein and CHAT protein, respectively. Fig. 4-*A,C* shows the relative protein expression obtained by densitometric analysis normalized to GAPDH. The nNOS-IR band density was strong in the ANP+saline rats, but significantly reduced in the corresponding ANP+octreotide group at 48 h (ANP+saline, 0.4992±0.0207; ANP+octreotide, 0.7204±0.0467; **, *P* = .001, n = 5; Fig. 4-*C*), but not at 24 h or 72 h. A second nNOS-IR band was observed, the size of which was reminiscent of the known nNOS splice variant nNOS-ß. The reduction of the CHAT-IR band density in the ANP+saline rats at 24 h compared with the ANP+octreotide rats could be considered as significant (ANP+saline, 0.4303±0.1086; ANP+octreotide, 0.5476±0.0972; *P* = .057; n = 5; Fig. 4-*B,D*).

## Discussion

In this study we demonstrated that damage of the enteric pathway of cholinergic and nitrergic innervation causes dysmotility of the small intestine within the first 72 h after the onset of ANP. Our results clearly showed that bolus applications of octreotide every 8 h in the following process of observation attenuated the functional abnormalities by ameliorating the injury of those enteric motor neurons. The present study provides for the first time evidence that octreotide may provide protection against ANP-related neuropathy.

Experimental and clinical studies have shown that acute pancreatitis induces intestinal dysmotility [1], [36], [37]. This is considered to lead to bacterial overgrowth and bacterial translocation, which are associated with the pathogenesis of pancreatitis-induced sepsis [4], [5], [6], [7]. The ENS is an autonomous entity that controls and coordinates motility, blood flow, and secretion throughout the gastrointestinal tract. Increasing evidence from animal models indicates that changes in the ENS are the underlying mechanisms for some motility disturbances [10], [11], [12], [13], [14]. The nitrergic and cholinergic nerves, which represent inhibitory and excitatory motor innervation, respectively, are usually the focus of studies of neuropathy [10], [11]. Our previous study showed that the pathogenesis of small intestinal paralysis in rats 24 h after ANP induction may be related to deficiencies in neuromuscular function [8]. To further explore the underlying mechanisms of ANP-induced neuropathy, we examined small intestinal motility within the first 72 h after ANP induction.

In the organ bath experiment, we observed that spontaneous ileal contractions declined significantly at 48 h in the ANP+saline rats, but reverted or climbed back up at 72 h. The receptor-mediated contractions to ACh in ANP rats was significantly lower at 24 h, but not at 48 h and 72 h. The complex and coordinated contractile activities performed by isolated segments of bowel depend on interactions between myogenic and local neural mechanisms. The changes in neuromuscular activities of the gut that occur under pathophysiological conditions have been the subject of intense animal research [10], [11], [12], [13], [14]. The morphological observations in the present study showed disruption of the structure of the PGP9.5-positive myenteric plexus at 24 h and significantly reduced numbers of nNOS and cholinergic neurons at all 3 time points. Both observations indicate obvious and indiscriminant damage of the myenteric plexus. The time course analysis suggested that the loss of inhibitory and excitatory enteric motor neurons occurred in the early stage of ANP and was attenuated as time went on, in line with the alteration of ileal contractions in our functional experiment. The reversibility of myenteric plexus disruption is a clear indication of neuronal plasticity within the ENS.

It is noteworthy that our previous study showed that the disturbance of myoelectric activity related to the damage of the interstitial cells Cajal during ANP gradually exacerbated [9]. The discrepancy in time course between myogenic and neural changes may provide an interesting issue that ANP-induced ileus, dependent on interplay of myogenic and neural regulation, may be postulated to progress from an early neuropathic form to a later myopathic form, as postulated by Owyang et al in a study of intestinal scleroderma [38]. Enteric neurons appear to be more vulnerable to injury caused by certain factors that occur in pathological states, such as inflammation and ischemia. However, they show a potential regenerative ability [12]. Thus, another mechanism by which, the myogenic factor, such as ICC, may be damaged by neurodegeneration or benefited by the following recovery of enteric neurons since the acquisition and maintenance of their adult phenotype are nerve-dependent [39]. These findings provide potential avenues for therapeutic intervention aimed at minimizing neuronal cell injury and the associated altered function of neuro-effector cell.

SOM inhibits growth hormone secretion. In the gastrointestinal tract, SOM is present in a subpopulation of descending interneurons that project caudally within the myenteric plexus [15]. The important physiological effects of SOM include regulation of intestinal fluid secretion, modulation of peristalsis, and enteric neurotransmission [15]. The diverse effects of SOM are mediated by specific, high-affinity, membrane-bound receptors termed SSTR_1–5_ [16]. Octreotide, a long-acting analogue of SOM, is an agonist with selectivity for the SSTR_2_ and SSTR_5_ receptors [16]. Most research suggests that the physiological effect of SOM on gastrointestinal transit is inhibitory [25], [26], [27]. And the site of action is largely believed to be at the level of the interneuronal enteric circuitry [29]. As a major coordinator of gastrointestinal activity, exogenous SOM may exert a serial of complex effects on intestinal motility in pathological states.

Another potentially beneficial property of octreotide is its antiinflammatory effect. The main mechanism has been considered to be the inhibition of proinflammatory cytokine and peptide release [16]. Under the conditions imposed by ANP, the systemic inflammatory responses may damage multiple organs including the intestine. The data from a recent clinical study show that SOM levels in the peripheral blood in all patients with acute pancreatitis were much lower than in healthy controls. Octreotide or somatostatin treatments could reduce the severity of histopathological injures of multiple organs, and could maintain the integrity of the intestinal mucosa by way of a mechanism that is associated with the inhibition of inflammatory mediators [21], [40]. Li recommended earlier administration of SOM or octreotide as soon as possible after the onset of acute pancreatitis may resolve some doubts of the relevancy of the experimental models to the clinical situations and consequently greatly benefit patients with acute pancreatitis, especially those at high risk [22].

Our organ bath experiments showed that, in contrast to the measurements in ANP+saline rats, the reduction of the contractile amplitude in the ANP+octreotide group didn't reach statistical significance and the tendency to decline reverted at 48 h, earlier than the ANP+saline group. Further, in contrast to the corresponding ANP+saline rats, the ANP+octreotide animals had significantly higher intestinal contractions at 24 h and 48 h. As for the contractile response to ACh, in contrast to the alterations of ANP+saline rats, no difference was observed between ANP+octreotide rats and their control at the 3 time points. When compared to ANP+saline rats, the ratios of contractile to ACh and to l-NNA in the ANP+octreotide rats were obviously higher at 24 h. The functional study implied that octreotide may have an influence on the pathways of inhibitory and excitatory motor innervation. The reduction in the number of neurons (including nNOS and cholinergic neurons) in the ANP+octreotide rats was greatly ameliorated at 24 h and 48 h, compared with the ANP rats. The protein expression of enteric neurons, furthermore, demonstrated that octreotide improved the impairment of CHAT neurons related to ANP at 24 h. Nevertheless, this similar protective effect to nNOS neurons was observed at 48 h. Taken together, our results suggests that octreotide could ameliorate the damage to enteric motor neurons in the early stage of ANP. However, the benefical effect seems to attenuate gradually over time.

An interesting observation was the upregulation and cellular internalization of SSTR_2_ in myenteric neurons. Pharmacological and clinical studies have suggested that SSTR_2_ is the important SSTR subtype involved with SOM-mediated inflammation and inhibition of GI motility [16]. Previous literature reported an upregulation of SSTR_2A_ expressed in myenteric neurons during the process of inflammation [16]. This coincides with our observation in ANP rats. This upregulation of SSTR_2_ may be explained as an antiinflammatory action of endogenous SOM, and it also suggests an enhanced inhibitory effect on the intestinal motor function in ANP rats. We, furthermore, found the diameter of SSTR_2_-positive cells in the ANP+octreotide group was smaller than in the control group. According to the published studies, the exogenous administration of SOM or its analogues may induce desensitization and internalization of several SOM receptor subtypes, including SSTR_2_ receptors [41], [42], which in turn resulted in a diminished effect of those reagent [43]. According to this evidence, we propose that enhanced SSTR_2_ expression related to an antiinflammatory action in the early stage of ANP indicates a protection against the neuropathy of ENS, and SSTR_2_ desensitization after a period of time of exposure to the exogenous octreotide may account for the attenuated protective effect for enteric motor neurons over time. Additional studies will be required to prove this hypothesis.

In summary, our results suggest that the pathogenesis of the ileus in the early stage of ANP may be related to the neuropathy of ENS, even though this injury is alleviated over time. Octreotide may ameliorate the severity of ileus by lessening the damage to inhibitory and excitatory motor innervation, even though this beneficial effect seems to attenuate gradually over time.
